# Metabolic heterogeneity of activated beige/brite adipocytes in inguinal adipose tissue

**DOI:** 10.1038/srep39794

**Published:** 2017-01-03

**Authors:** Yun-Hee Lee, Sang-Nam Kim, Hyun-Jung Kwon, James G. Granneman

**Affiliations:** 1 College of Pharmacy, Yonsei University, Incheon, 21983, South Korea; 2 Center for Integrative Metabolic and Endocrine Research, Wayne State University School of Medicine, Detroit, MI, 48201, USA

## Abstract

Sustained β3 adrenergic receptor (ADRB3) activation simultaneously upregulates fatty acid synthesis and oxidation in mouse brown, beige, and white adipose tissues; however, the cellular basis of this dual regulation is not known. Treatment of mice with the ADRB3 agonist CL316,243 (CL) increased expression of fatty acid synthase (FASN) and medium chain acyl-CoA dehydrogenase (MCAD) protein within the same cells in classic brown and white adipose tissues. Surprisingly, in inguinal adipose tissue, CL-upregulated FASN and MCAD in distinct cell populations: high MCAD expression occurred in multilocular adipocytes that co-expressed UCP1+, whereas high FASN expression occurred in paucilocular adipocytes lacking detectable UCP1. Genetic tracing with UCP1-cre, however, indicated nearly half of adipocytes with a history of UCP1 expression expressed high levels of FASN without current expression of UCP1. Global transcriptomic analysis of FACS-isolated adipocytes confirmed the presence of distinct anabolic and catabolic phenotypes, and identified differential expression of transcriptional pathways known to regulate lipid synthesis and oxidation. Surprisingly, paternally-expressed genes of the non-classical gene imprinted network were strikingly enriched in anabolic phenotypes, suggesting possible involvement in maintaining the balance of metabolic phenotypes. The results indicate that metabolic heterogeneity is a distinct property of activated beige/brite adipocytes that might be under epigenetic control.

Adipocytes are specialized cells that store, mobilize and metabolize lipids to maintain energy homeostasis^1^. Historically, adipocytes have been categorized into white and brown adipocytes, and their respective anabolic and catabolic functions in lipid metabolism have been well-characterized^2^. White adipocytes store excess energy as triglyceride (TG) from which free fatty acids are mobilized to meet systemic energy demands. Brown adipocytes generate heat within brown adipose tissue by uncoupling ATP synthesis from mitochondrial fatty acid oxidation^3^. In addition to classic brown and white adipocytes, an additional metabolic phenotype, the beige or brite (for ‘brown in white’) adipocyte, has been identified in certain adipose tissue depots, such as the inguinal fat pad of mice^4^,^5^,^6^. Under basal conditions, beige/brite adipocytes express very low levels of prototypical brown adipocyte proteins like UCP1; however, acute adrenergic receptor activation strongly upregulates expression of brown adipocyte proteins in depots containing beige/brite cells, although the absolute levels achieved within the tissue as a whole are much lower than that observed in classic BAT^7^. Whether activated beige/brite adipocytes are a distinct cell phenotype apart from classic brown adipocytes is controversial and presently unresolved.

While it is well known that adrenergic stimulation increases catabolic metabolism in brown and white adipose tissues^3^,^8^, it is not widely appreciated that chronic activation also upregulates *de novo* fatty acid synthesis, which allows cells to meet oxidative demand with enhanced synthesis^9^,^10^,^11^. The mechanisms that couple and segregate anabolic and catabolic pathways, however, are not understood.

We previously reported that chronic pharmacological activation of ADRB3 simultaneously upregulates fatty acid synthesis and oxidation in brown, beige/brite, and white adipose tissues^9^. In this study, we examined upregulation of anabolic and catabolic gene expression at the cellular level using immunohistochemistry, genetic tracing, and fluorescence activated cell sorting (FACS). Our results indicate that beige/brite adipocytes adopt distinct anabolic and catabolic phenotypes in response to adrenergic activation. Global transcriptomic analysis of FACS-isolated adipocytes confirmed the existence of distinct anabolic and catabolic phenotypes and identified differential expression of known and novel transcriptional regulators, including non-classical imprinted gene network. The presence of anabolic and catabolic phenotypes within the inguinal adipose tissue organ suggests the potential for functional interactions between cells that synthesize and export fatty acids and those that oxidize them.

## Results

### Chronic β3 adrenergic activation simultaneously upregulates fatty acid synthesis and oxidation in brown, beige/brite and white adipose tissues

**Figure 1:**
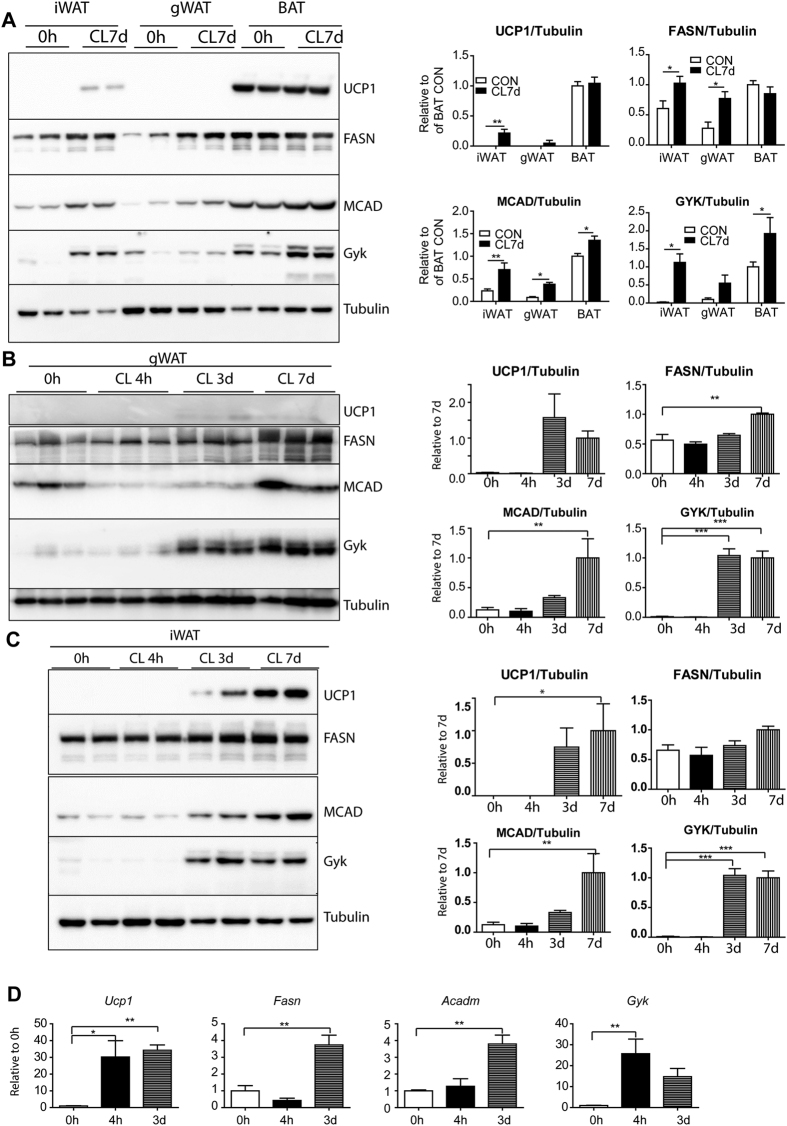
Chronic β3 adrenergic activation simultaneously upregulates fatty acid synthesis and oxidation in brown, beige and white adipose tissues. (**A**–**C**) Immunoblot analysis and quantification of UCP1, FASN, MCAD, and GYK expression in iWAT, gWAT and BAT from mice treated with CL up to 7 days. Tubulin was used as a loading control (n = 4 per condition; mean ± SEM), *P < 0.05, **P < 0.01, ***P < 0.001). (**D**) Quantitative PCR analysis of Ucp1, Fasn, Acadm, and Gyk in iWAT of mice treated with CL for up to 3 days (n = 4 per condition; mean ± SEM), *P < 0.05, **P < 0.01).

We previously reported that chronic ADRB3 activation simultaneously upregulates de novo fatty acid synthesis and oxidation in brown (interscapular), beige (inguinal), and white (gonadal) adipose tissues^9^. To compare the induction of proteins involved in thermogenesis, FA mitochondrial oxidation, FA synthesis and re-esterification by ADRB3 activation, we examined expression levels of UCP1, medium-chain specific acyl-CoA dehydrogenase (MCAD), fatty acid synthase (FASN), and GYK (glycerol kinase) in inguinal WAT (iWAT), gonadal WAT (gWAT) and interscapular BAT from mice treated with CL for 7 days. As shown in Fig. 1A, BAT contained highest levels of UCP1, FASN, MCAD, and GYK under basal and activated conditions among adipose tissue depots examined. Seven days of CL treatment significantly increased GYK and MCAD expression in BAT. CL treatment significantly increased MCAD expression in iWAT by 3 fold, resulting in levels that were 80% of those observed in control BAT. CL treatment also upregulated FASN and GYK expression by 2-fold and 9-fold, respectively. Seven days of CL treatment strongly induced UCP1 expression in iWAT, reaching 20% of that observed in control BAT. In gWAT, CL treatment significantly induced MCAD, FASN and GYK, but not UCP1. These observations indicate that induction of catabolic/anabolic gene expression occurs similarly in classic brown and white adipocytes, as well as in beige adipose depots, regardless of UCP1 status.

Next, we monitored the time course of the catabolic and anabolic gene expression in iWAT and gWAT during CL treatment (Fig. 1B–D). iWAT and gWAT had similar patterns of each protein and mRNA expression, showing significant induction UCP1, FASN, MCAD, and GYK in iWAT by day 3 (Fig. 1B–D).

### CL treatment increases FASN and MCAD in distinct adipocyte population

**Figure 2:**
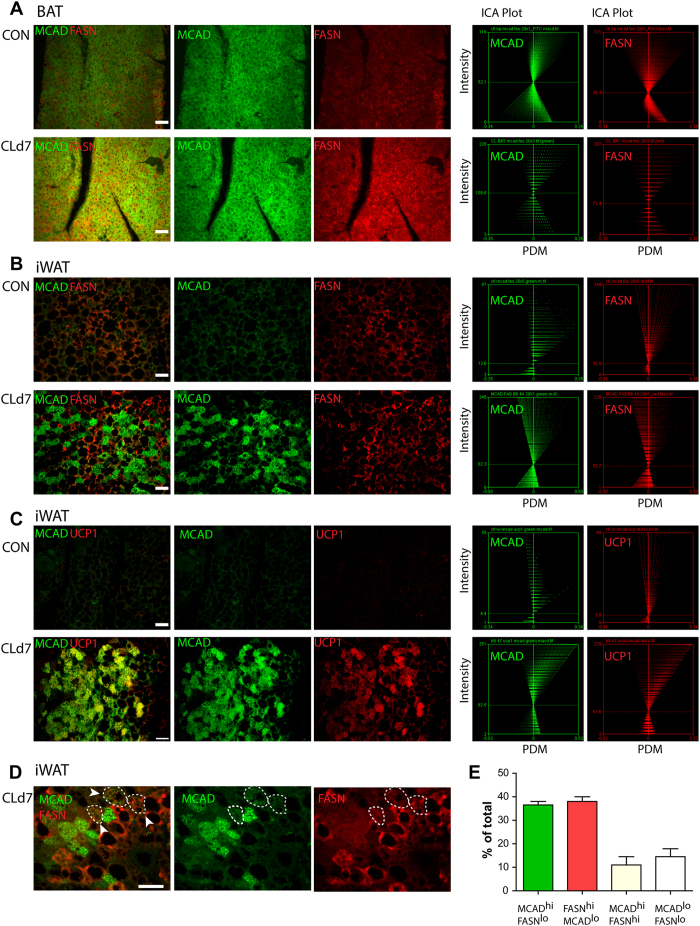
CL treatment increases FASN and MCAD in distinct adipocyte population. (**A**) Representative images of paraffin sections of BAT double-stained for MCAD and FASN, and intensity colocalization analysis of the images. (**B**,**C**) Representative images of paraffin sections of iWAT double-stained for MCAD and FASN, or MCAD and UCP1, and intensity colocalization analysis of the images. The axes on the plots are the product of the differences from the mean (PDM) values on the x-axis and the red or green intensity on the y-axis PDM = (red intensity-mean red intensity) X (Green intensity-mean green intensity). (**D**) High magnification images of paraffin sections of iWAT double stained for MCAD and FASN. Circled areas (with arrowheads) indicate MCAD, FASN double positive adipocytes. (**E**) Quantitation of images of paraffin sections of iWAT double stained for MCAD and FASN. (n = 4, total 200 > adipocytes per condition) Size bars = 40 μm.

To study cellular basis of induction of catabolic and anabolic gene expression, we examined the expression of MCAD, FASN and UCP1 by immunohistochemistry. In BAT, MCAD and FASN were uniformly expressed throughout the parenchyma of the tissue (Fig. 2A). Interestingly, in iWAT, high levels of MCAD and FASN were observed in separate adipocyte populations (Fig. 2B). We calculated co-localization of each protein by using automatic image analysis developed by Li *et al*.^12^. If two proteins are colocalized, then the product of the differences from the mean (PDM: (red intensity-mean red intensity) × (green intensity-mean green intensity)) values will be positive. Conversely, if the staining patterns are segregated, PDM values will be negative. The intensity correlation quotient (ICQ) is based on the non-parametric sign-test analysis of PDM values (ratio of the number of positive PDM values to the total number of pixel values); thus, ICQ between 0 and −0.5 indicates segregated staining and ICQ between 0 and +0.5 indicates correlated staining. Intensity correlation analysis^12^ demonstrated MCAD and FASN immunofluorescence was segregated (ICQ = −0.021 ± 0.02, mean ± SEM, n = 4), whereas elevated UCP1 and MCAD staining was observed in the same cells (ICQ = 0.232 ± 0.04, mean ± SEM, n = 4. ICQ values from analysis of UCP1/MCAD double staining were significantly higher than those from MCAD/FASN double staining (two-tailed t-test, p = 0.0036) (Fig. 2B,C). Occasionally we observed cells that expressed moderate levels of both FASN and MCAD (Fig. 2D). To confirm the expression at cellular level, we manually counted cells that expressed high levels of FASN and MCAD (above mean values of each fluorescence intensity of the field) and found that 48.4 ± 2.3% and 40.6 ± 3.3% of cells were FASN^hi^, MCAD^lo^ and FASN^lo^, MCAD^hi^ adipocytes, respectively (Fig. 2E). Only 8.48 ± 2.39% of adipocytes were positive for both proteins.

### Adipocytes with history of UCP1 expression can become either FASN^hi^ anabolic adipocytes or MCAD^hi^ catabolic adipocytes

**Figure 3:**
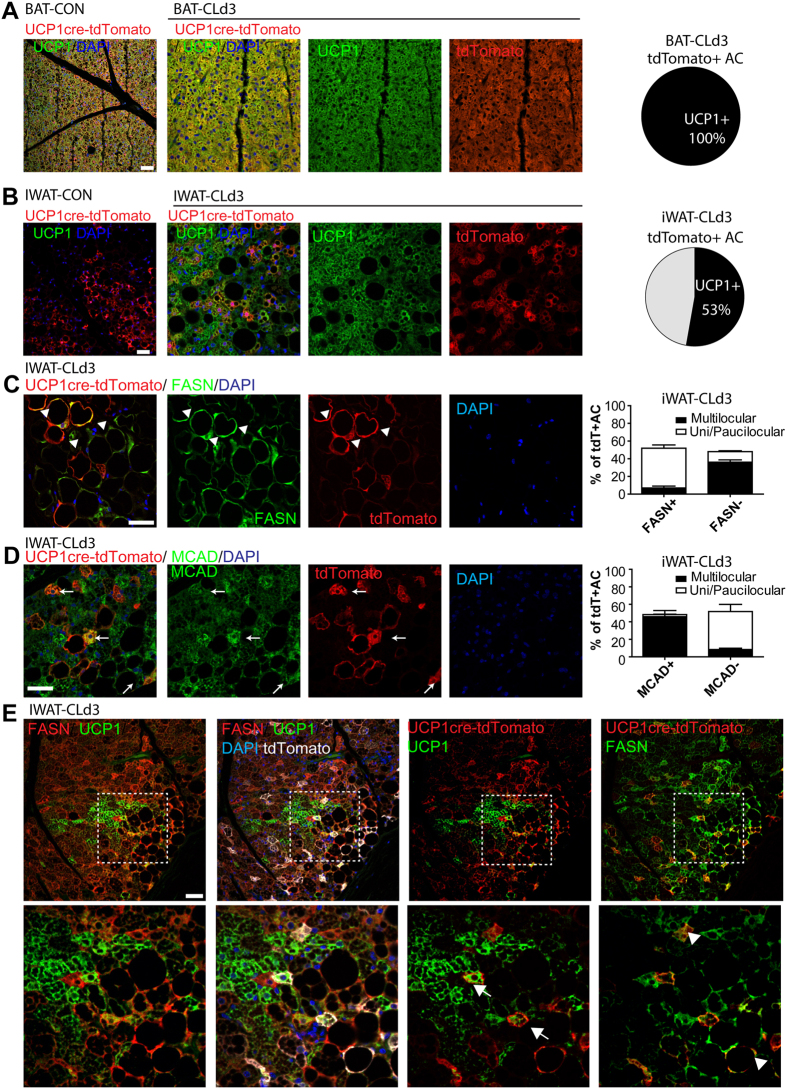
Adipocytes with UCP1 expression history become FASN^hi^ and MACD^hi^ adipocytes. (**A**,**B**) Representative images of paraffin sections of BAT (**A**) and iWAT (**B**) double-stained for UCP1 and tdTomato, and quantification of UCP1+tdTomato+/tdTomato+ adipocytes. (**C**) Representative images of paraffin sections of iWAT double-stained for FASN and tdTomato, and quantification of populations of tdTomato+ adipocytes distinguished by cellular morphology and FASN staining (n = 3 mice (>100 tdTomato+ adipocytes per animal). Arrows indicate tdTomato+FASN+ adipocytes. (**D**) Representative images of paraffin sections of iWAT double-stained for MCAD and tdTomato, and quantification of population of tdTomato+ adipocytes distinguished by cellular morphology and MCAD staining (n = 3 mice (>100 tdTomato+ adipocytes per animal). Arrows indicate tdTomato+MCAD+adipocytes. (**E**) Representative images of paraffin sections of iWAT triple-stained for FASN, UCP1 and tdTomato. Arrows indicate tdTomato+UCP1+ adipocytes whereas arrowheads indicate FASN+tdTomato+ adipocytes. Size bars = 40 μm.

Previous reports suggested that there are distinct cell lineages that can become beige/brite adipocytes^13^,^14^,^15^. To test if anabolic and catabolic adipocytes were derived from the beige/brite adipocyte lineage, we genetically traced adipocytes having a history of UCP1 expression. In this experiment, double transgenic mice that expressed Cre recombinase under the control of the Ucp1 promoter and Cre-responsive tdTomato reporter (floxed stop codon) were treated with CL for 3 days and FASN and MCAD expression was analyzed by immunohistochemistry. UCP1-cre uniformly induced expression of the tdTomato reporter in brown adipocytes in the BAT parenchyma of control mice and mice treated with CL for 3 days (Fig. 3A). IWAT from control mice contained numerous tdTomato+ adipocytes lacking detectable UCP1 expression, indicating the presence of beige/brite adipocytes that once expressed UCP1, but current levels were beneath the sensitivity of immunofluorescence (Fig. 3B). We noticed that CL-induced UCP1 expression did not always coincide with tdTomato expression (i.e., UCP1+/tdTomato – cells were detected) (Fig. 3B). The appearance of UCP1+/tdTomato- cells might be due to differences in the timing of Cre expression of the transgene (versus native genes), the speed of recombination, or expression of tdTomato from the ROSA26 locus. Therefore, we restricted our histological analysis to cells that expressed tdTomato. Immunofluorescence analysis indicated that tdTomato+ adipocytes could be nearly equally divided into multilocular, MCAD^hi^ cells and paucilocular, FASN^hi^ cells (Fig. 3C–E). Of cells with definitive history of Ucp1 expression, 95 ± 1% (mean ± SD) of MCAD^hi^ cells were multilocular and 5 ± 1% were paucilocular/unilocular, whereas 14 ± 8% of FASN^hi^ cells were multilocular and 86 ± 8% were pauci/unilocular. Collectively, these data indicate that adipocytes with a history of UCP1 expression can become anabolic or catabolic adipocytes in the presence of adrenergic activation.

### Transcriptomic profiling of catabolic and anabolic adipocytes

**Figure 4:**
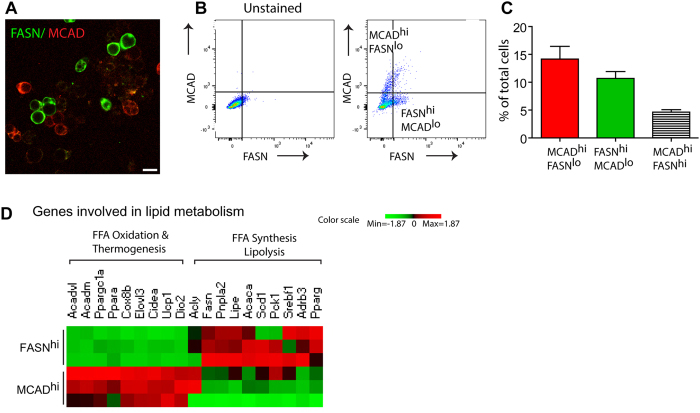
Isolated FASN^hi^ and MCAD^hi^ adipocytes in iWAT expressed genes involved in anabolic and catabolic lipid metabolism, respectively. (**A**) Immunostaining of FASN and MCAD in adipocytes fractionated from iWAT of mice treated with CL for 3 days. Size bar = 20 μm. (**B**) Flow cytometry analysis of FASN and MCAD expression in adipocytes isolated from iWAT treated with CL for 3 days. (**C**) Quantification of MCAD^hi^FASN^lo^ (MCAD^hi^) and FASN^hi^MCAD^lo^ (FASN^hi^) and MCAD^hi^FASN^hi^ adipocytes. (**D**) Heatmap of expression levels of genes involved in lipid metabolism in FASN^hi^MCAD^lo^(FASN^hi^) and MCAD^hi^FASN^lo^ (MCAD^hi^) adipocytes. Adipocytes from iWAT were fractionated and sorted for FASN and MCAD expression. A red-green color scale depicts normalized expression levels (based on Z-scores) in FPKM values.

To further confirm heterogeneity under conditions where cell borders are distinct, we performed immunofluorescence staining of FASN and MCAD in adipocytes fractionated from iWAT of mice treated with CL for 3 days. As shown in Fig. 4A, high levels of MCAD and FASN expression were detected in distinct adipocytes. These results suggested that it would be possible to isolate distinct populations using newly developed techniques for staining of intracellular antigens and isolation by FACS for transcriptome analysis by RNA-seq^16^. Thus, to more fully characterize anabolic and catabolic phenotypes, we FACS-isolated FASN^hi^ and MCAD^hi^ adipocytes and performed global transcriptome analysis. Consistent with histological examination, FACS analysis identified distinct populations FASN^hi^ and MCAD^hi^ cells, with relatively few cells (4.62 ± 0.46%) simultaneously expressing high levels of the anabolic and catabolic markers (Fig. 4A–C). In planned comparisons based on previous work^9^, we observed higher expression of the catabolic genes Ppara, Acadvl, Gyk and Acadm in MCAD^hi^ cells, whereas expression of anabolic genes Pparg, Srebf1, and Fasn was upregulated in FASN^hi^ cells (Fig. 4D). Surprisingly, FASN^hi^ cells also expressed higher levels of β3 adrenergic receptors (Adrb3), adipose triglyceride lipase (ATGL, Pnpla2), and hormone-sensitive lipase (HSL, Lipe), indicating that synthesis might be coupled to a greater degree of lipolysis, but not fatty acid oxidation (Fig. 4D). Additional differentially-regulated genes included brown adipocyte and thermogenic markers UCP1, Dio2, Elovl3, Cidea and Cox8b (Fig. 4D). Although expression of these brown adipocyte markers was higher in MCAD^hi^ adipoctyes, substantial levels of these transcripts were also detected in FASN^hi^ cells, indicating underlying metabolic flexibility.

**Figure 5:**
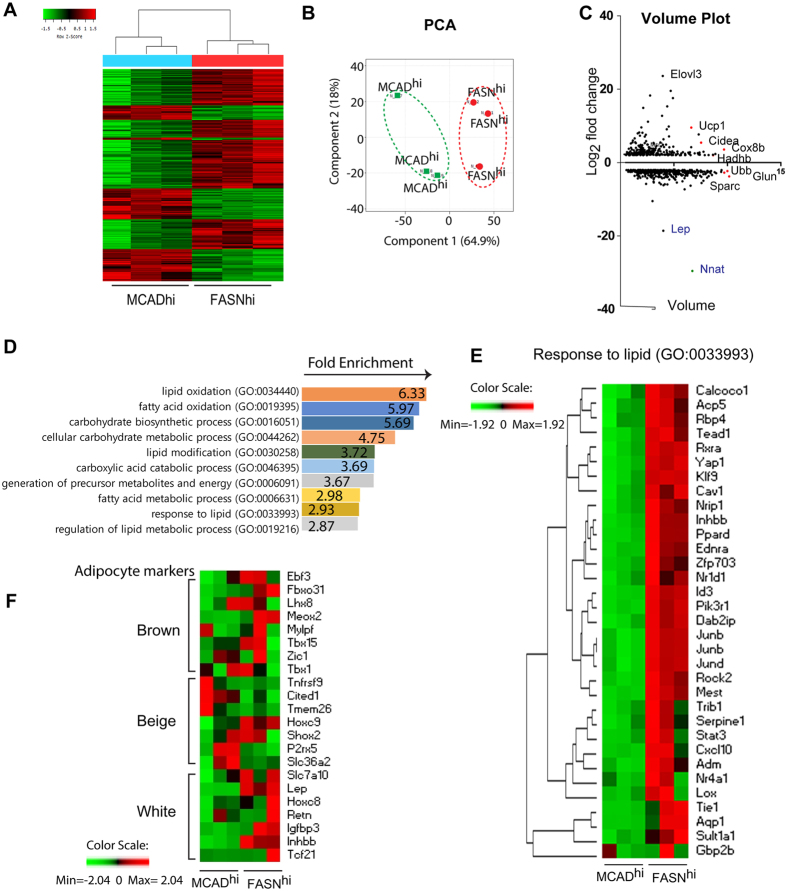
Transcriptomic characterization of FASNhi and MCADhi adipocytes isolated from iWAT of mice treated with CL. (**A**) Heat map of 653 genes differentially regulated in MCAD^hi^ cells compared to FASN^hi^ cells from RNAseq data (2 > fold changes, p < 0.05). (**B**) PCA analysis of transcriptome of FASN^hi^ and MCAD^hi^ adipocytes. The first two PCA components is shown. Distinct two clusters of Red (FASN^hi^) and geen (MCAD^hi^) were distinguished by component 1. (**C**) Volume plot of differentially regulated genes in MCAD^hi^ adipocytes compared to FASN^hi^ with fold change ≥ 2 (p < 0.05). X axis: volume = square root (control normalized value (FASN^hi^ group) X test normalized value (MCAD^hi^ group), Y axis: log_2_ (Fold Change). (**D**) Examples of GO terms enriched in the differentially regulated genes between FASN^hi^ and MCAD^hi^ adipocytes with Fold change ≥ 2 (p < 0.05). Fold enrichment is displayed for GO term (p < 0.05). (**E**) Heat map of the differentially regulated genes belong to GO 00339931.F. Heat map of planned comparison of genes known for white/beige/brown adipocyte markers.

In total, the expression of 653 genes differed by >2 fold between FASN^hi^ and MCAD^hi^ populations (Fig. 5A). The differentially-expressed genes were clustered by principal components analysis (PCA) and categorized by gene ontology (GO) (Fig. 5B,D). This analysis indicated that genes involved in fatty acid oxidation (GO 0019395, Acaa2, Acadm, Acadvl, Decr1, Ehhadh, Etfdh, Fabp3, Hacl1, Hadha, Hadhb, Irs2, Lep, Plin5, Por, Ppara, Ppard, Ppargc1a, Slc27a2, p = 3.7E-26) were expressed at higher levels in MCAD^hi^ adipocytes. Genes involved in the response to lipid stimuli (GO0033993) were also differentially regulated between two groups (Fig. 5E). Importantly, proposed beige adipocyte markers (CD137, TBX1, TBX15 TMEM26, Slc36a2, Slc7a10, or P2RX5)^5^,^17^,^18^ and determination factors (PRDM16 and EBF2)^19^ (Fig. 5F) were similarly expressed in the two phenotypes.

**Figure 6:**
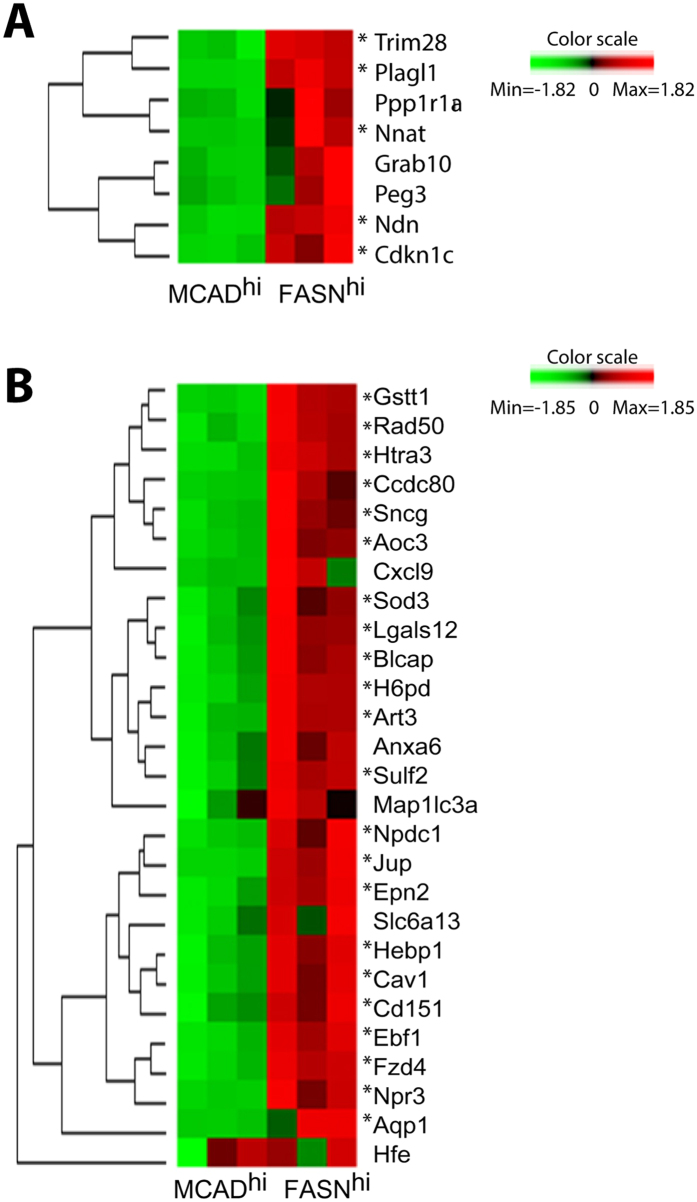
Distinct imprinted gene expression patterns in isolated FASN^hi^ and MCAD^hi^ adipocytes of iWAT from mice treated with CL for 3 days. Heat map and hierarchical clustering of RNA-seq data for imprinted genes (**A**) and Nnat-related network (**B**) (* = raw p < 0.05).

Apart from well-established genes involved in lipid synthesis and oxidation, FASN^hi^ and MCAD^hi^ cells differentially expressed several well-known imprinted genes that are paternally-expressed (maternally silenced) and have been linked to lipid metabolism and variations in body fat content^20^. TRIM28 was recently found to regulate stochastic variations in body fat content involving a non-classical imprinted gene network (IGN)^21^. Neuronatin (Nnat) is a member of the IGN that was expressed at much higher levels in FASN^hi^ cells (Figs 5C and 6A). Moreover, 22 of the 27 genes (24/27 including trends) that were identified as being co-regulated with Nnat^22^ were significantly (p < 0.0001) overexpressed in FASN^hi^ cells (Fig. 6B). Interestingly, knock down of Nnat in inguinal adipocytes was recently found to upregulate expression of catabolic genes, including Cox8b and Ucp1^23^. In addition, mesoderm-specific transcript (Mest/Peg1), which positively associated with adipocyte triglyceride accumulation^24^,^25^ was expressed at much higher levels in FASN^hi^ cells.

## Discussion

It is well known that thermogenic stimuli increases catabolic metabolism in adipose tissues that involves upregulation of genes mediating mitochondrial biogenesis and fatty acid oxidation. Chronic adrenergic activation also upregulates *de novo* fatty acid synthesis, which allows cells to meet oxidative demand with enhanced synthesis; however, the basis of this metabolic plasticity is not understood. Previous studies demonstrated that ADRB3 stimulation simultaneously upregulates genes involved in catabolic and anabolic lipid metabolism in whole tissues. In the current study, we used immunohistochemical analysis to probe the cellular localization of anabolic and catabolic proteins that are upregulated in response to ADRB3 stimulation.

In classic brown and white adipose tissue depots, adrenergic stimulation upregulated anabolic and catabolic enzymes in the same cell type. In contrast, upregulation of FASN and MCAD/UCP1 expression in inguinal adipose tissue was largely restricted to distinct adipocyte populations. Importantly, lineage tracing with UCP1-cre demonstrated that FASN^hi^ cells can arise from adipocytes that once expressed Ucp1. Indeed, cells with a history of UCP1 expression, as indicated by cre-mediated recombination, were almost equally divided between FASN^hi^ and MCAD^hi^/UCP1^hi^ adipocytes.

These results indicate that adipocytes within inguinal adipose tissue adopt distinct metabolic phenotypes in the face of persistent adrenergic activation. While differences in the catabolic states of adipocytes are known to occur in the absence and presence of thermogenic activation^3^,^8^, the induction of distinct anabolic and catabolic cellular phenotypes during adrenergic activation was prominently observed only in inguinal fat, and could be a distinguishing feature of beige/brite adipocytes.

Global mRNA profiling of sorted FASN^hi^ and MCAD^hi^ adipocytes provided independent confirmation of distinct catabolic and anabolic phenotypes. First, flow cytometry demonstrated distinct cell populations of FASN^hi^ or MCAD^hi^, with relatively few cells exhibiting high levels of each. As expected, MCAD^hi^ cells expressed high levels of genes involved in mitochondrial biogenesis and fatty acid oxidation. These cells also expressed higher levels of brown adipocyte markers, including Ucp1, Dio2, and Cidea, consistent with the multilocular phenotype observed. In contrast, FASN^hi^ cells expressed higher levels of genes involved in lipogenesis, fatty acid uptake, and lipolysis, suggesting that one function of these cells might be to supply neighboring oxidative cells with a steady supply of fatty acids. In this regard, FASN^hi^ cells expressed higher levels of Angptl2 and Angptl4, which would suppress lipoprotein lipase activity and might facilitate trafficking of LDL-derived fatty acids to oxidative phenotypes. While our results clearly establish the presence of phenotypic heterogeneity using morphological, protein, and gene expression criteria, the direct impact on metabolism must await the development of tools capable of evaluating metabolic fluxes *in situ* at single cell resolution.

It is important to note that FASN^hi^ cells expressed significant levels of Ucp1 mRNA and other brown adipocyte markers, albeit at levels lower than MCAD^hi^ cells. Furthermore, proposed brown/beige markers were similarly expressed in the FASN^hi^ and MCAD^hi^ cells. These observations, along with UCP1-cre tracing, indicate that these metabolic phenotypes represent inherent plasticity of a single beige/brite cell type. It is presently unclear whether this heterogeneity reflects developmental plasticity or perhaps the ability of cells to switch between phenotypes during sustained stimulation, as suggested by the small population of MCAD^hi^, FASN^hi^ adipocytes. It is important to note that this heterogeneity would be missed by approaches that evaluate whole tissues or rely solely on a history of Ucp1 expression (e.g., Ucp1-Cre).

Global profiling also provided insights into possible mechanisms for cellular plasticity. For example, differential expression of Pparg, Rxra, and Srebpf provide plausible transcriptional pathways for expanding *de novo* lipogenesis in the FASN^hi^ cells. Similarly, Ppara is a well-known regulator of mitochondrial biogenesis and fatty acid oxidation, and has been shown to play an important role in catabolic remodeling of white adipose tissue^26^. Unexpectedly, we found that genes of the non-classical IGN were strikingly differentially-expressed in anabolic and catabolic phenotypes. Although the function of this pathway is quite complex and requires further study^21^,^22^, recent work indicates that the non-classical IGN plays an important role in epigenetic regulation of body fat content, with global loss of function mutations increasing the frequency of individuals with elevated body mass^21^. Our data demonstrate that a paternally-expressed gene network involving Nnat and Mest is overexpressed in FASN^hi^ cells relative to MCAD^hi^ cells and might play a role in phenotype stability. In this regard, knock down of Nnat expression was recently shown to promote catabolic gene expression in cultured inguinal adipocytes^23^, consistent with the phenotypes observed in the present study. Mest is also expressed exclusively from the paternal allele, and its expression level in white adipocytes has been found to predict the magnitude of fat accumulation when genetically identical mice are fed standard or high fat diets^24^. Furthermore, adipocyte-specific overexpression of Mest in mice increases fat cell size^27^, whereas knockout suppresses adipocyte hypertrophy induced by high fat feeding^28^. Whether variations in Nnat and Mest expression contribute to cellular heterogeneity within adipose tissues is an important question for future studies.

## Methods

### Animals

All animal experiments were conducted in strict compliance with the guidelines for humane care and use of laboratory animals as specified by the Ministry of Food and Drug Safety, and the National Institutes health Guide for Care and Use of Laboratory Animals. All animal protocols were approved by the Institutional Animal Care and Use Committees of Yonsei and Wayne State Universities. UCP1-Cre mice (B6.FVB-Tg(Ucp1-cre)1Evdr/J, Stock # 024670) and LoxP-Stop-LoxP-tdTomato mice (B6.Cg-Gt(ROSA)26Sortm9(CAG-tdTomato)Hze/J, Stock #007914) were purchased from the Jackson laboratory and crossed to produce double transgenic mice. Genotyping was performed as described previously^13^,^14^. C57BL/6 mice (5–6 wk old, male) were purchased from the Jackson Laboratory (Bar Harbor, ME) and Orient Bio (Gyeonggi-Do, South Korea). Mice were fed a standard chow diet. For continuous β3 adrenergic receptor stimulation, mice were infused with CL316,243 (Sigma, St. Louis, MO) (0.75 nmol/h) using osmotic pumps (Alzet, Cupertino, CA; 1007D) for up to 7 days.

### Immunohistochemistry and immunocytochemistry

Adipose tissue was processed for histological sections, and 5-μm-thick paraffin sections were subjected to immunohistochemical analysis, as previously described^13^.

To evaluate co-localization of immunostaining, three random 200X images were analyzed for an individual sample, and biological triplicates or quadruplicates of each condition were used. Intensity correlation analyses were performed as described by Li *et al*.^12^. Intensity correlation analysis (ICA) plots and color scatter plots were generated by the ImageJ ICA plugin. Intensity Correlation Quotient (ICQ) values were used to determine dependent or segregated distribution of the staining. Alternatively, each fluorescent intensity of cells were measured by using ImageJ. At least 100 adipocytes per an individual sample were analyzed, and biological triplicates of each condition were used to determine percentage of cells that express the proteins above the mean value of the fluorescence intensity of the field. All quantification of histologic samples was carried out as blind analyses.

### Gene expression

For quantitative PCR analysis, RNA was extracted using TRIzol reagent (Invitrogen) and converted into cDNA by using high-capacity cDNA synthesis kit (Applied Biosystems, Waltham, MA). Quantitative PCR was performed using SYBR Green Master Mix (Applied Biosystems) and ABI StepOne PLUS (Applied Biosystems) for 45 cycles, and the fold change for all the samples was calculated by the comparative cycle-threshold (Ct) method (i.e., 2−ΔΔCt method). Peptidylprolyl isomerase A was used as the housekeeping gene for mRNA expression analysis. cDNAs were amplified using previously described primers^9^.

### Western blot analysis

Protein extracts were prepared as previously described^9^. Western blot analysis was performed using primary antibodies against UCP1 (mouse, Abcam; or rabbit Alpha Diagnostic International), FASN (rabbit, Cell Signaling), MCAD (mouse, Santa Cruz Biotechnology), GYK (rabbit, Cell Signaling) and tubulin (mouse, Santa Cruz Biotechnology) and secondary anti-mouse, and anti-rabbit horseradish peroxidase antibodies (Cell Signaling Technology, Danvers, MA), as described previously (**17**). The blots were visualized with SuperSignal West Dura Substrate (Pierce-Invitrogen).

### Adipocyte fractionation and flow cytometry

Dissociated adipocyte fractions were isolated from three independent preparations of inguinal white adipose tissue (iWAT) of three pooled mice treated with CL for 3 days, as previously described^13^. Adipocytes were fixed with 4% paraformaldehyde (Electron Microscopy Science, Hatfield, PA) on ice for 15 min, permeabilized with 0.1% saponin (Sigma) and processed for intracellular immunostaining^16^. The antibodies used for immunochemical detection were anti-FASN (rabbit, 1:50; Cell Signaling) and anti-MCAD antibody (mouse, 1:50; Santa Cruz). The secondary antibodies used were goat anti-rabbit-PE or APC (1:200, ThermoFisher) and goat anti-mouse-Alexa Fluor 488 (1:200, ThermoFisher). The omission of primary antibody was used as a negative control. All buffers used in immunostaining contained 0.0025% RNasin Plus RNase Inhibitor (Promega)^16^,^29^. Cell sorting was performed using BD FACSAria III (BD Biosciences, San Jose, CA). 5 × 10^5^ cells of each condition were sorted and then centrifuged at 1000 × g for 5 min at 4 °C. RNA crosslinking was reversed by incubation with proteinase K solution at 56 °C for 1 h and then RNA was isolated using miRNeasy FFPE isolation kit (Qiagen), as described previously^29^. For RNAseq analysis, cDNA libraries were constructed with the TruSeq mRNA library kit using 1 ug of total RNA. The libraries were quantified using qPCR according to the qPCR Quantification Protocol Guide and subjected to 100 nt paired-end sequencing using the Illumina HiSeq2000.

To estimate expression levels, the RNA-Seq reads were mapped to the genome of Mus musculus using TopHat^30^, which is capable of reporting split-read alignments across splice junctions and determined using Cufflinks software^31^ in default options. The reference genome sequence of Mus musculus and annotation data were downloaded from the Pepper Genome Platform (PGP) ftp site (http://passport.pepper.snu.ac.kr/?t=PGENOME).

### Gene expression analysis of mRNAseq transcriptomics data

For mRNA expression, the transcript counts in isoform level were calculated, and the relative transcript abundances were measured in FPKM (Fragments Per Kilobase of exon per Million fragments mapped) using Cufflinks. We excluded transcripts with zeroed FPKM values more than one for total samples. We added 1 with FPKM value of the filtered transcript to facilitate log2 transformation. Filtered data was transformed by logarithm and normalized by quantile normalization method. For each transcript, calculated fold change between case and control. Differentially expressed transcripts were determined by adjusting |fold change| ≥ 4 in more than at least one of total comparisons.

For gene-GO term enrichment analysis, biologically gene functional annotation analysis for DEG list was performed using DAVID tool (http://david.abcc.ncifcrf.gov/)^32^ to assess biological function of the large gene set. All data analysis and visualization of differentially expressed genes was conducted using R 3.1.2 (www.r-project.org).

### Statistical analysis

Statistical analyses were performed with GraphPad Prism 5 software (GraphPad Software, La Jolla, CA). PCA were performed with XLStat software (Addinsoft, New York, NY) to detect the common variations between variables and to visualize clusters of correlated observations. Heatmap and hierarchical clustering were generated by PermutMatrix program with Euclidean distance for dissimilarity and complete linkage for aggregation criteria. The graphical representation is based on Z-scores. Normalized expression values for each genes (rows) are standardized to have mean 0 and standard deviation 1^33^.

Data are presented as means ± SEM. Statistical significance between two groups was determined by unpaired t-test or Mann-Whitney U-test, as appropriate. For qPCR and immunoblot analysis, comparison among multiple groups was performed using a one-way ANOVA or two-way ANOVA, with Bonferroni post hoc tests to determine the relevant p values. For planned comparisons of RNAseq data, unpaired t-test were used to determine raw p values.

## Additional Information

**How to cite this article**: Lee, Y.-H. *et al*. Metabolic heterogeneity of activated beige/brite adipocytes in inguinal adipose tissue. *Sci. Rep.* **7**, 39794; doi: 10.1038/srep39794 (2017).

**Publisher's note:** Springer Nature remains neutral with regard to jurisdictional claims in published maps and institutional affiliations.
